# Automatic radiation-free evaluation of Cobb angle for spinal curvature based on fringe projection profilometry and deep learning technology

**DOI:** 10.1007/s43390-025-01270-1

**Published:** 2026-01-15

**Authors:** Chi-Kuang Feng, Ching-Jung Hung, Yen-Ju Chen, Pei-Yu Su, Guan-Ting Liu, Cheng-Yang Liu

**Affiliations:** 1 Department of Orthopaedics and Traumatology, Taipei Veterans General Hospital, Taipei, Taiwan; 2 Department of Biomedical Engineering, National Yang Ming Chiao Tung University, Taipei, Taiwan; 3 Medical Device Innovation and Translation Center, National Yang Ming Chiao Tung University, No. 155, Sec. 2, Linong Street, Beitou District, Taipei City, Taiwan

**Keywords:** Idiopathic scoliosis, Fringe projection, Deep learning, Noninvasive detection

## Abstract

**Objective:**

Pediatric scoliosis is the most prevalent spinal disorder, often leading to abnormal curvature and deformation of the spine. Early detection is essential for timely intervention, particularly in growing adolescents. In this study, we present a novel, fully automated, radiation-free method for Cobb angle evaluation, combining fringe projection profilometry with deep learning technologies.

**Materials and methods:**

A three-dimensional reconstruction of the participant’s back surface is achieved using a seven-step phase-shifting algorithm. Convolutional neural networks are then utilized to extract asymmetry features from the 3D surface and predict the Cobb angle, a key clinical indicator of scoliosis severity. A total of 48 participants clinically diagnosed with scoliosis based on radiographic imaging were recruited from the hospital.

**Results:**

The experimental results demonstrate a strong correlation between the predicted and actual Cobb angles, with a correlation coefficient of 0.94 and a coefficient of determination of 0.8796 during Adam’s forward bend test. The mean time required from scanning to Cobb angle prediction is approximately 3.3 s.

**Conclusions:**

The proposed evaluation method exhibits excellent discriminative capability and shows significant potential as an alternative to the traditional scoliometer for large-scale Cobb angle screening programs in schools.

## Introduction

Idiopathic scoliosis is a three-dimensional (3D) spinal deformity predominantly affecting adolescents aged 10–18 years [1]. Diagnosis is based on the Cobb method, defined as a coronal curvature > 10° in the absence of congenital, neuromuscular, or secondary causes [2]. Severe scoliosis can result in functional disability, pain, cardiopulmonary compromise, and early spinal degeneration [3]. Radiography remains the standard for assessing curve severity, monitoring progression, and guiding treatment [4]. The Cobb angle, measured between the upper and lower end vertebrae, is the gold standard for quantifying deformity [5–7]. Screening tools aim to identify scoliosis before significant progression. The scoliometer, used with Adam’s forward bend test, achieves sensitivity and specificity of 83.3% and 86.8% [8, 9], but requires manual placement, is time-consuming, and correlates only moderately with the Cobb angle (*r* = 0.677) [5]. Moiré topography projects contour lines to visualize asymmetries caused by spinal rotation [10–12], but requires perpendicular projection, limiting use during forward bending, and has false-positive rates of 32–60% [13–15]. Recent advances integrate 3D depth-sensing with predictive algorithms to address these limitations [16–19]. Such systems capture back-surface topography in ~ 1.5 s, providing rapid, noninvasive assessment with reliable correlation to radiographic Cobb angles. Notably, optical screening systems have received medical device approval in Japan, demonstrating their potential for safe, radiation-free, and efficient Cobb angle detection in school-based programs.

Deep learning has shown remarkable success in extracting complex patterns from large datasets [20]. Convolutional neural networks (CNNs), comprising convolutional, pooling, and fully connected layers, are particularly effective for feature extraction [21]. CNN-based methods have been widely applied to surface detection and classification tasks [22–24], including the detection, classification, and segmentation of spinal tumors and metastases [25, 26]. For scoliosis, CNNs using medical images or Moiré topography have demonstrated potential in curve assessment [27–29], yet remain limited in accurately localizing vertebral deformities and predicting Cobb angles. Fringe projection profilometry (FPP) offers a complementary approach for 3D surface measurement [30–32]. In FPP, digital fringe patterns are projected onto a surface, and the phase of the resulting deformed patterns encodes depth information. Phase-shifting algorithms extract the phase distribution, enabling 3D reconstruction via phase–height relationships and stereo triangulation [33]. FPP provides non-contact, high-speed, full-field imaging with operational simplicity, making it suitable for applications from industrial inspection to biomedical characterization [34–38]. Its adaptability to confined spaces and varying lighting conditions further supports its potential for scoliosis assessment in clinical and school-based settings [39].

In this study, we propose a radiation-free evaluation method to address key challenges associated with Cobb angle assessment. A fully automated, high-speed digital fringe projection profilometry system is employed to rapidly capture three-dimensional characteristics of the patient’s back surface. The reconstructed 3D back profiles are subsequently used to train a CNN for regression-based Cobb angle prediction, enabling accurate and rapid estimation without the need for external markers or complex preparation procedures. By avoiding delays inherent to conventional surface imaging workflows, the proposed approach streamlines data acquisition and analysis. Given that idiopathic scoliosis predominantly develops during adolescence, reliable and low-risk assessment methods are particularly important during early disease stages. The specific objective of this study is to develop and validate a fully automated, radiation-free system for estimating the Cobb angle from back surface morphology and to evaluate its accuracy and feasibility in a proof-of-concept clinical setting by comparison with radiographic measurements.

## Methods

**Fig. 1:**
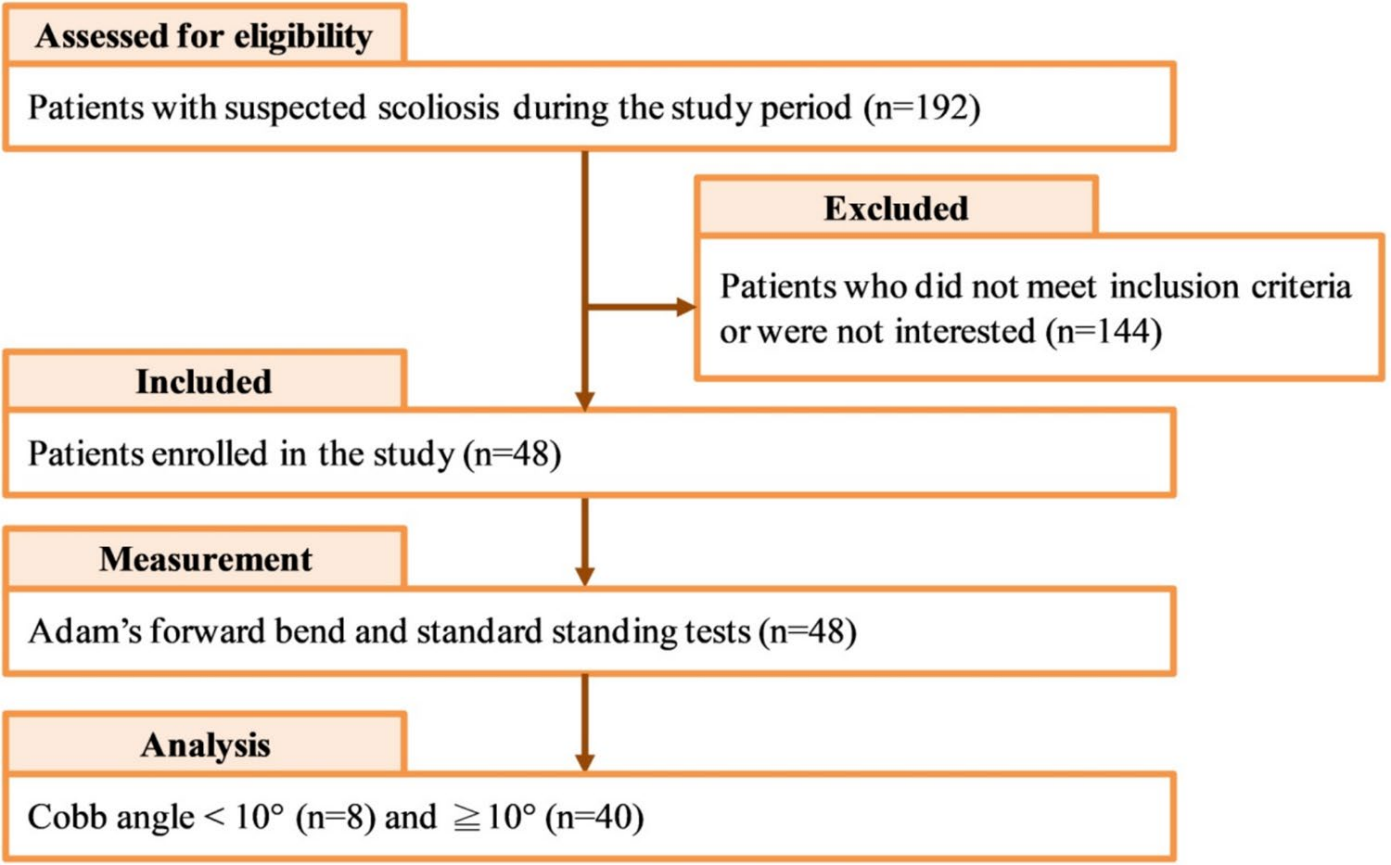
CONSORT flowchart of this study

The experimental protocols were approved by the Institutional Review Board (IRB) of the hospital. Informed consent was obtained from the parents or legal guardians of all pediatric participants, in accordance with institutional guidelines. The inclusion criteria were as follows: (1) age between 7 and 18 years; (2) confirmed scoliosis based on radiographic imaging; (3) no prior history of brace treatment; and (4) willingness and ability to provide written informed consent or assent. Exclusion criteria included the presence of neuromuscular, syndromic, or congenital scoliosis. A total of 48 participants (12 males and 36 females) were recruited from the hospital for analysis (Fig. 1). Upon obtaining consent, participants' medical records were reviewed to compare the diagnostic accuracy of the proposed system with the current standard of care. Collected clinical data included age, gender, body mass index (BMI), and Cobb angle assessed from digital radiographs. The mean age of participants was 12.3 years (range, 7–18 years), with a mean BMI of 18.6 kg/m^2^ (range, 13.4–27.1 kg/m^2^), and a mean Cobb angle of 21.3° (range, 2°–78°). Scoliosis is clinically defined as a curve > 10°, but cases with Cobb angles both above and below this threshold were included to train the CNN model based on surface morphology. Under clinical supervision, participants performed standardized Adam’s forward bend and standing tests with the upper body exposed. The 3D back surface was then captured using the proposed imaging system for model development and evaluation.

**Fig. 2:**
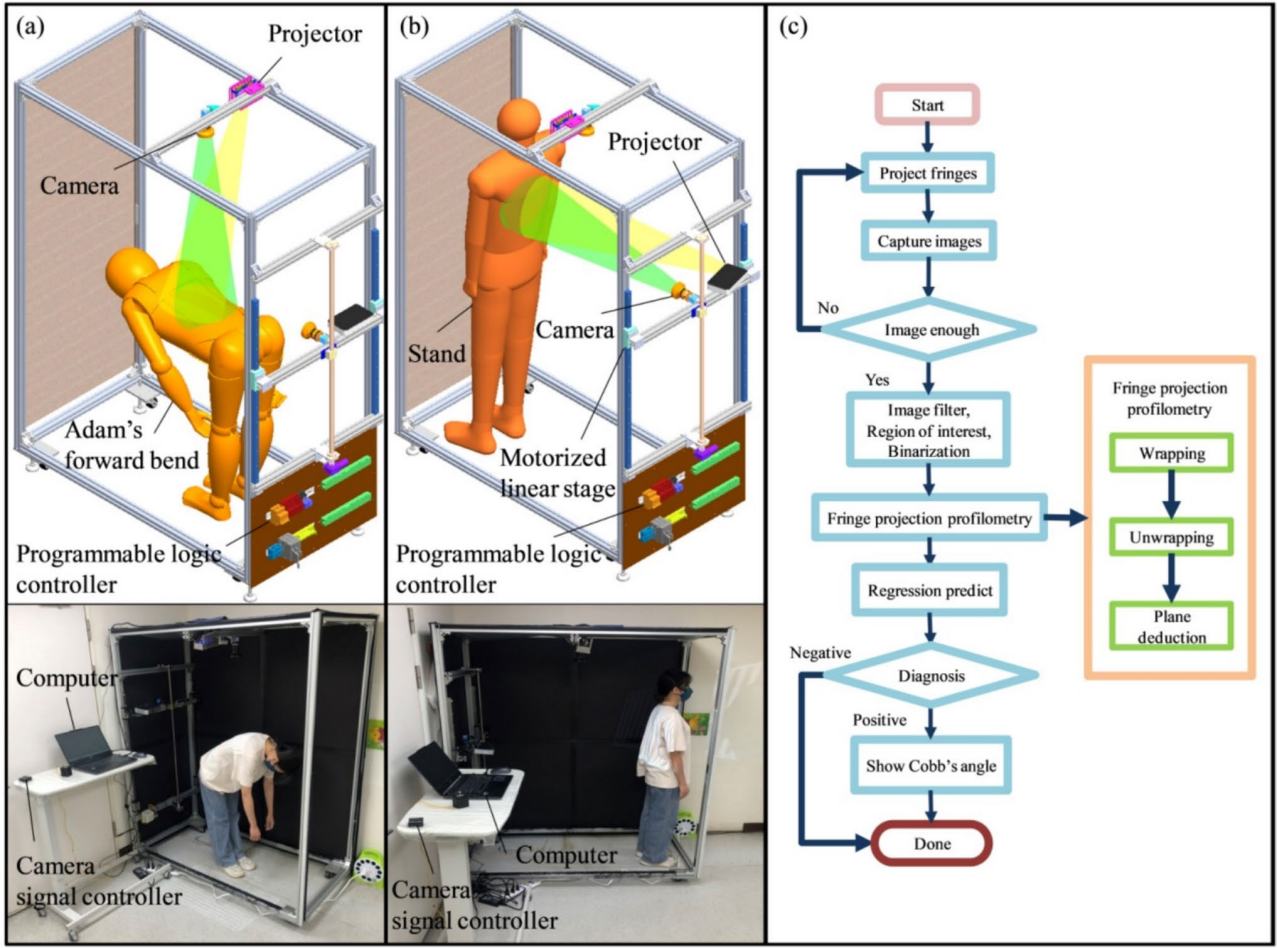
Experimental setups and photographs of automatic radiation-free evaluation system for Cobb angle with **a** Adam’s forward bend test and **b** stand test. **c** Flowchart of three-dimensional surface imaging, processing, and prediction for the human back

To achieve high reliability and repeatability in the detection of Cobb angle, accurate measurement of back surface deformities is essential. In this study, a seven-step phase-shifting fringe projection technique is employed to rapidly reconstruct the 3D profile of the patient’s back. Figure 2 illustrates the experimental setup of the proposed radiation-free evaluation system for Cobb angle. The imaging system consists of two digital light processing (DLP) projectors (Texas Instruments DLP3010EVM-LC), two monochrome cameras (The Imaging Source DMK 33UP5000), a motorized linear stage, and a laptop computer. The projectors provide a brightness of 125 lumens and a resolution of 1280 × 720 pixels, while the cameras operate at a frame rate of 60 frames per second with a resolution of 2592 × 2048 pixels. Each projector–camera pair is used to capture the 3D shape of the back during either Adam’s forward bend test or a standard standing posture assessment. The evaluation system is deployed in the outpatient pediatric orthopedic clinic at the hospital and can be fully set up within a few minutes. Both the imaging components and the subjects are situated inside a compact, light-controlled darkroom to eliminate interference from ambient light and ensure measurement accuracy. During image acquisition, participants were required to remove upper-body clothing to ensure accurate capture of back-surface morphology. To protect patient privacy, the entire imaging system was enclosed by black curtains, creating a private measurement space. All measurements were performed while the participant stood within this enclosed area, thereby preventing any exposure of personal privacy.

Figure 2c illustrates the flowchart for the 3D surface imaging, processing, and prediction procedure for the patient's back. Fringe patterns are generated by a computer program and projected onto the patient's back via the projector. Both the projector and camera are mounted on a motorized linear stage, enabling precise alignment of the imaging system with the centerline of the patient's back. As the fringe patterns are projected, they are deformed according to the surface contour of the back. These deformed fringe images are captured by the camera and stored on the computer for subsequent processing. Captured grayscale images are first subjected to binarization, transforming them into black-and-white images to enhance contrast [40]. Gaussian blur filtering is then applied as a low-pass filter to eliminate background noise and suppress high-frequency artifacts in the images [41]. Following image optimization, phase maps are calculated from the deformed fringe patterns using a phase-shifting algorithm [31, 42]. Specifically, a seven-step phase-shifting technique, an extension of the conventional three-step method, is employed to achieve high-resolution 3D surface imaging, significantly enhancing the smoothness of the reconstructed back shape [31]. The wrapped phase map representing the target surface is initially obtained through the seven-step phase-shifting calculation. Subsequently, the quality-guided path method is applied for phase unwrapping, providing high reliability in demodulating the wrapped phase to real-world dimensions [31]. Full 3D geometric reconstruction of the patient's back is thus achieved through a sequential process of fringe projection, phase estimation, phase unwrapping, and geometric transformation. During data acquisition, patients are guided to adopt postures corresponding to Adam’s forward bend test and standard standing test, with their upper bodies exposed for optimal imaging. The resulting 3D back reconstructions are compiled to form the training dataset for the CNN model. Following model training, the system outputs the predicted Cobb angle derived from the deep learning algorithm, enabling efficient and accurate scoliosis evaluation.

**Fig. 3:**
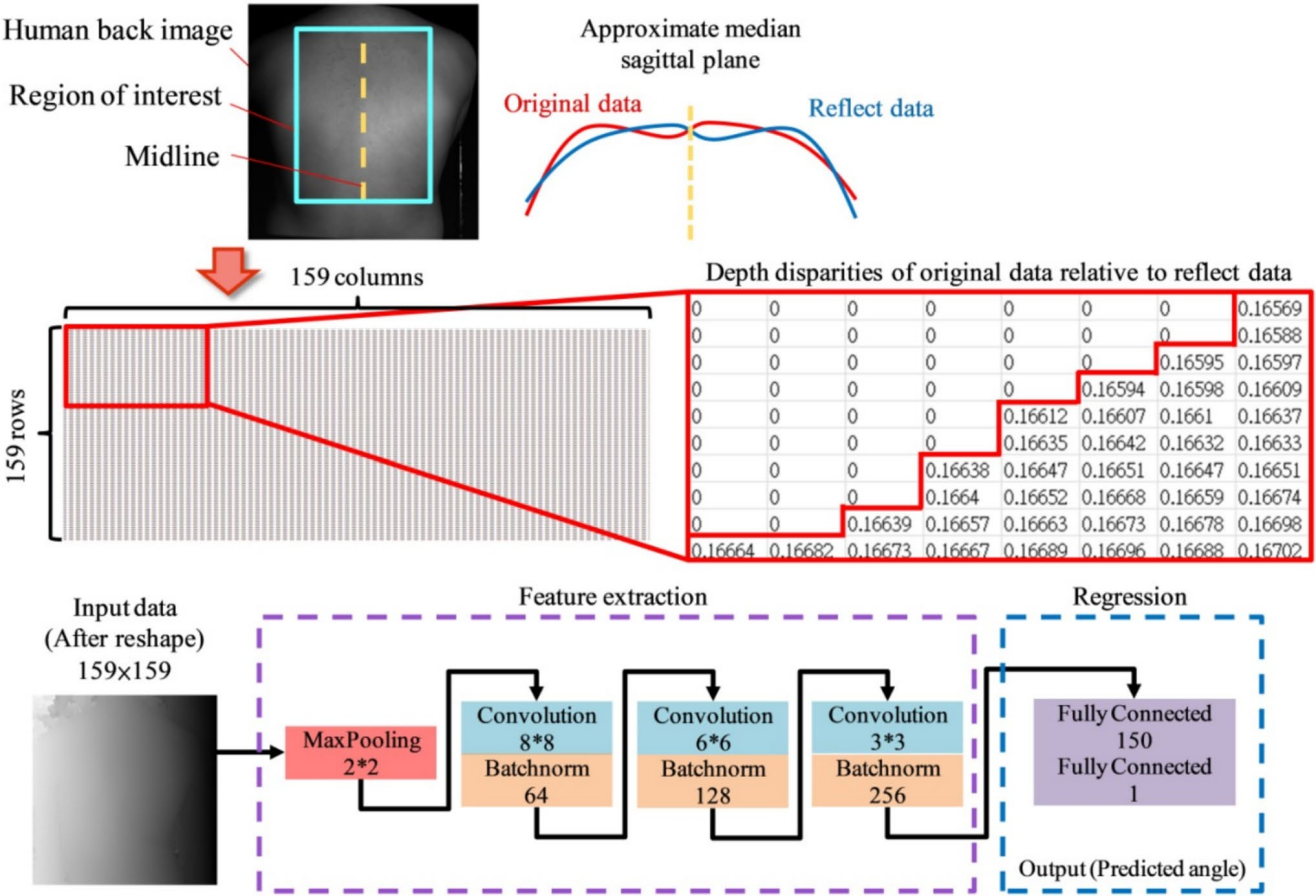
Overview of the convolutional neural network architecture for evaluating the asymmetry degree of the human back

In this study, a CNN model is employed to extract and analyze the morphological contours of patients' backs. Figure 3 illustrates the CNN architecture designed to evaluate the degree of asymmetry in the patient's back. The back contour is captured using a seven-step phase-shifting fringe projection technique, resulting in high-resolution 3D surface data. Region of interest (ROI) is defined as a rectangular area extending from the waist to both shoulders, aligned along the approximated midsagittal plane. To quantify back asymmetry, mirrored data are generated by reflecting the original 3D surface data across the sagittal plane. The spatial differences between the original and mirrored datasets are used as input features for the CNN model, effectively capturing the altitude disparities between the right and left sides of the back relative to the sagittal plane. The depth disparity data are organized into comma-separated values (CSV) files with 159 rows and 159 columns, where non-relevant areas are zero-padded. A total of 48 original CSV files were collected from the participants to construct the initial dataset. To enhance model robustness and generalization, data augmentation techniques were applied. Specifically, augmented datasets were created by shifting, rotating, and zooming the lateral regions from the waist to the upper, middle, and lower sections, generating nine additional CSV files for each original file. In total, 432 augmented CSV files were produced, from which 346 files were randomly selected for the training set and 86 files for the validation set. The CNN architecture includes an initial image augmentation layer, followed by three convolutional layers, each integrated with a MaxPooling layer to reduce spatial dimensions while preserving key features. The network concludes with a Huber loss function as the output layer, chosen for its robustness against outliers in the dataset. For training, the model was configured with a learning rate of 0.001, a batch size of 8, and 500 epochs, utilizing the Adam optimizer for adaptive learning. The training and validation processes were conducted on a high-performance workstation equipped with an Intel Core i9-10920X CPU, an NVIDIA GeForce RTX 3080 Ti GPU, and 128 GB of RAM.

**Fig. 4:**
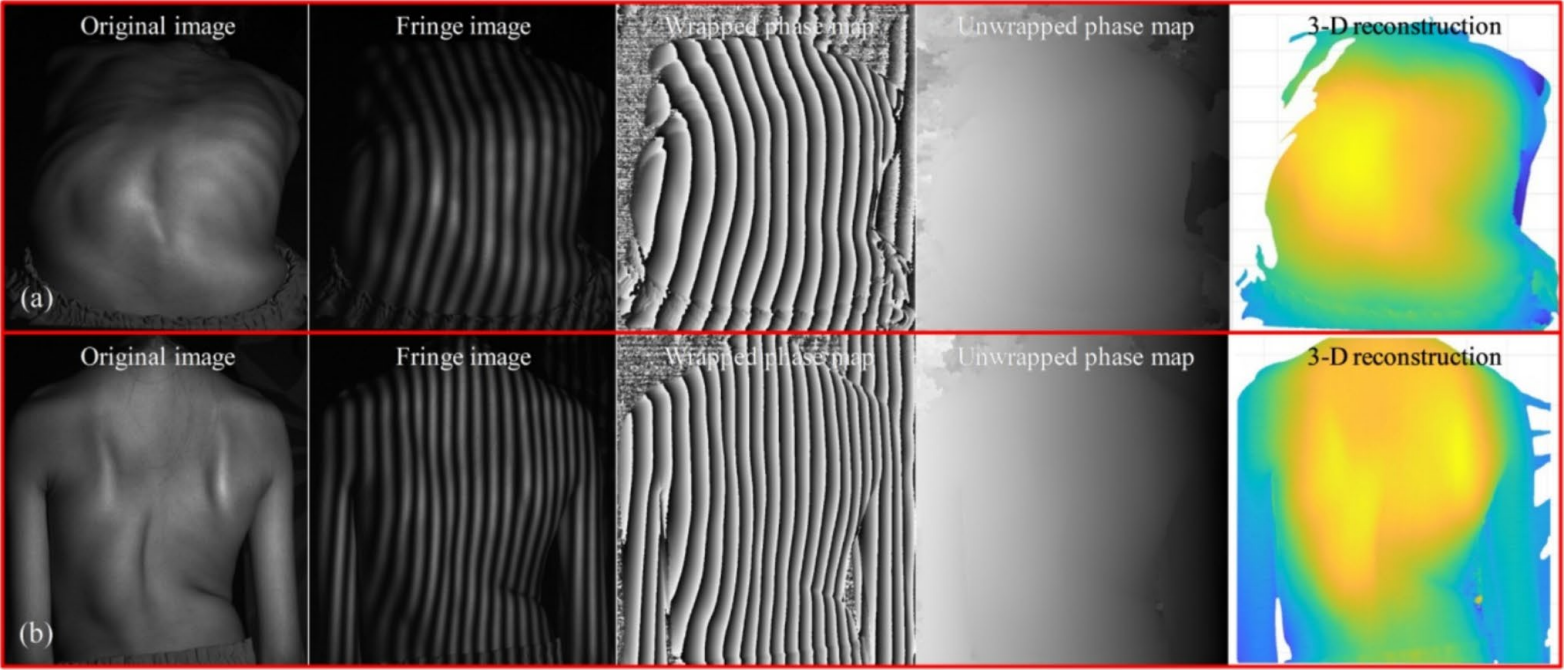
Process sequence of phase-shifting measurements of the human backs for **a** Adam’s forward bend test and **b** stand test

The radiation-free evaluation system for Cobb angle assessment was developed to validate the performance of a seven-step phase-shifting fringe projection algorithm. As shown in Fig. 4, sequential phase-shifting measurements were performed during both the Adam’s forward bend test and the standing test. Seven computer-generated fringe patterns were projected onto the patient’s back using a high-speed DLP projector, allowing rapid pattern switching. Each deformed fringe pattern was captured by a camera synchronized with the projector and stored for subsequent processing. The wrapped phase map was calculated from the acquired fringe images using the seven-step phase-shifting algorithm, in which surface deformation information was encoded in the phase distribution. Since the wrapped phase is inherently periodic with a period of 2π, a quality-guided path algorithm was applied to perform phase unwrapping, yielding a continuous phase map. The unwrapped phase was then converted into a three-dimensional back-surface model through geometric phase-to-height calibration. For visualization purposes, color texture mapping was applied to the reconstructed surface. To enable spinal alignment estimation using fringe projection profilometry, a dataset of 3D back reconstructions was collected for deep learning model training and evaluation. During the initial scoliosis assessment, all participants underwent standard radiographic examination to confirm the diagnosis. Each patient contributed a standing whole-spine radiograph and a corresponding three-dimensional back reconstruction, which were used for comparative analysis in subsequent modeling.

## Results

**Fig. 5:**
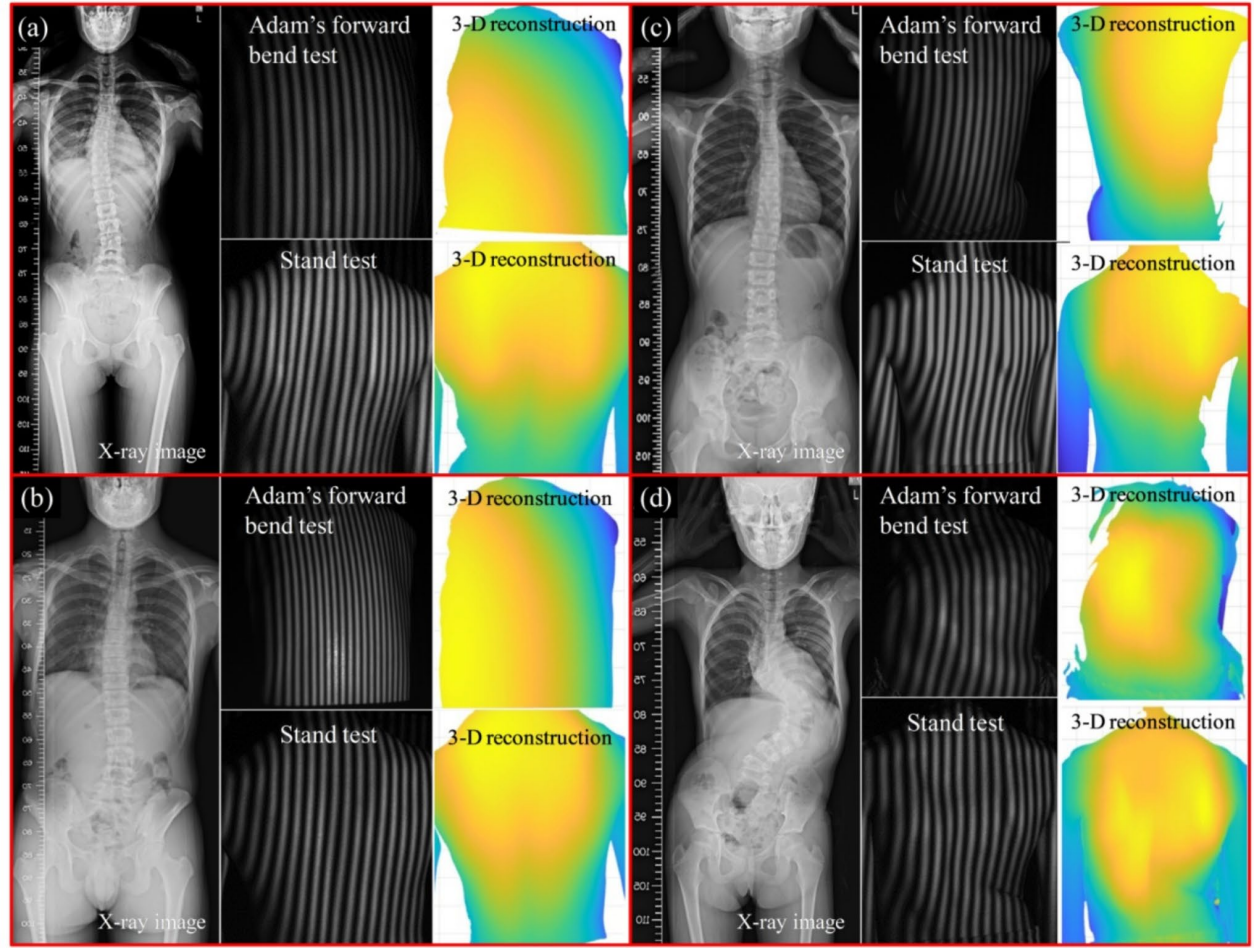
Phase-shifting measurements of the human backs for **a** thoracic, **b** lumbar, **c** thoraco-lumbar, and **d** combined scoliosis

**Fig. 6:**
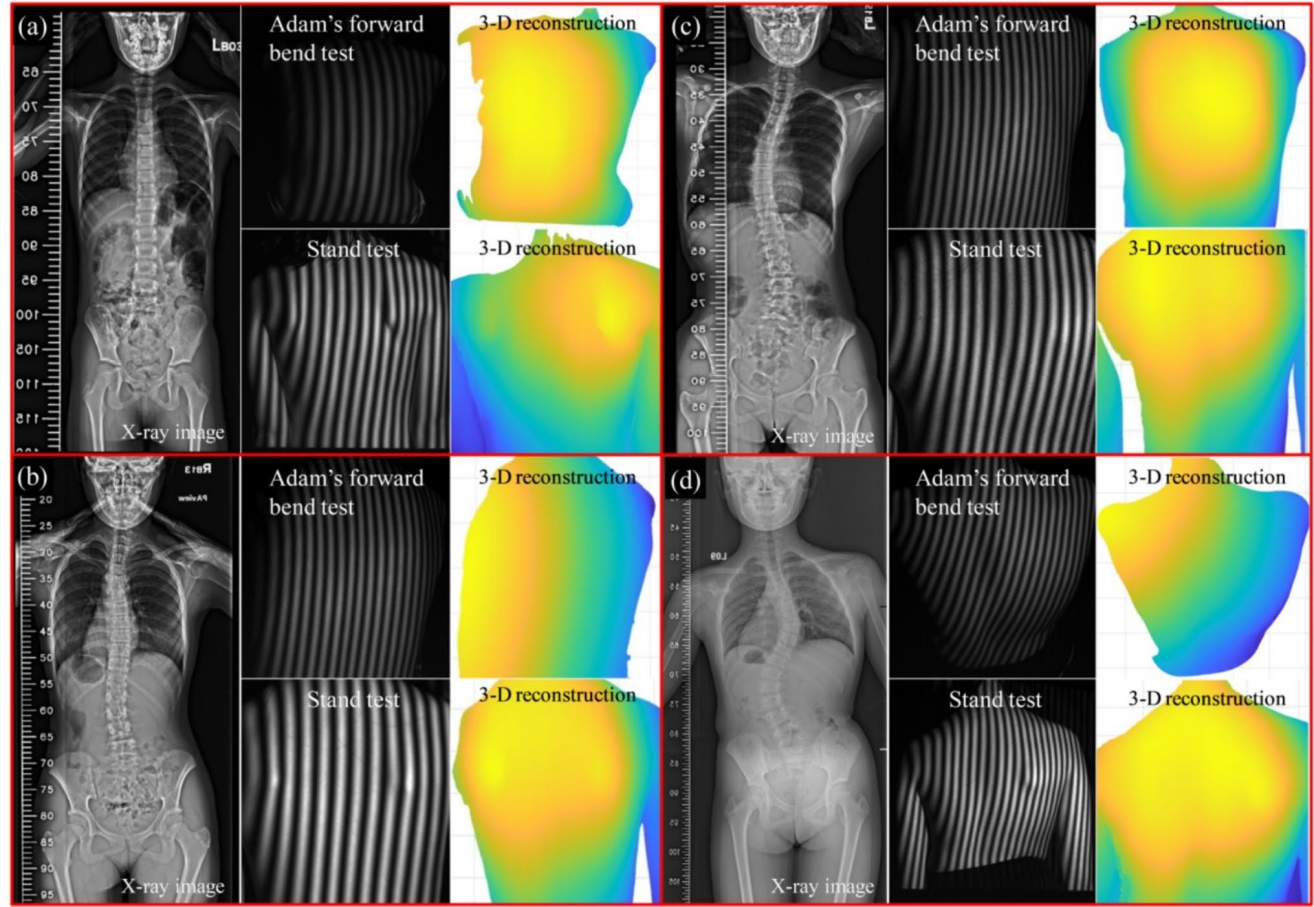
Phase-shifting measurements of the human backs for Cobb angles of **a** 4.5°, **b** 14.7°, **c** 27°, and **d** 59°

Posture was found to have a notable influence on measurement characteristics. During the Adam’s forward bend test, back-surface asymmetry was more prominently observed in the mid-back region, whereas in the standing posture, asymmetry tended to shift toward the upper back. These posture-dependent differences were consistently observed across subjects. Figure 5 presents phase-shifting measurements and 3D reconstructions of patients with thoracic, lumbar, thoraco-lumbar, and combined scoliosis, captured during both Adam’s forward bend and standing tests. Reconstructions from both postures demonstrated sufficient quality for convolutional neural network training. Importantly, the degree of asymmetry in the 3D reconstructions qualitatively correlated with scoliosis severity as defined by the Cobb angle. Figure 6 illustrates representative cases with Cobb angles of 4.5°, 14.7°, 27°, and 59°, showing a clear trend in which surface asymmetry increased proportionally with Cobb angle magnitude. This effect was most pronounced in the middle and upper back regions for both postures. Furthermore, the apex location of spinal curvature identified in radiographs corresponded closely with the regions of maximal deviation in the 3D reconstructions, underscoring the validity of surface-based imaging for Cobb angle assessment.

**Fig. 7:**
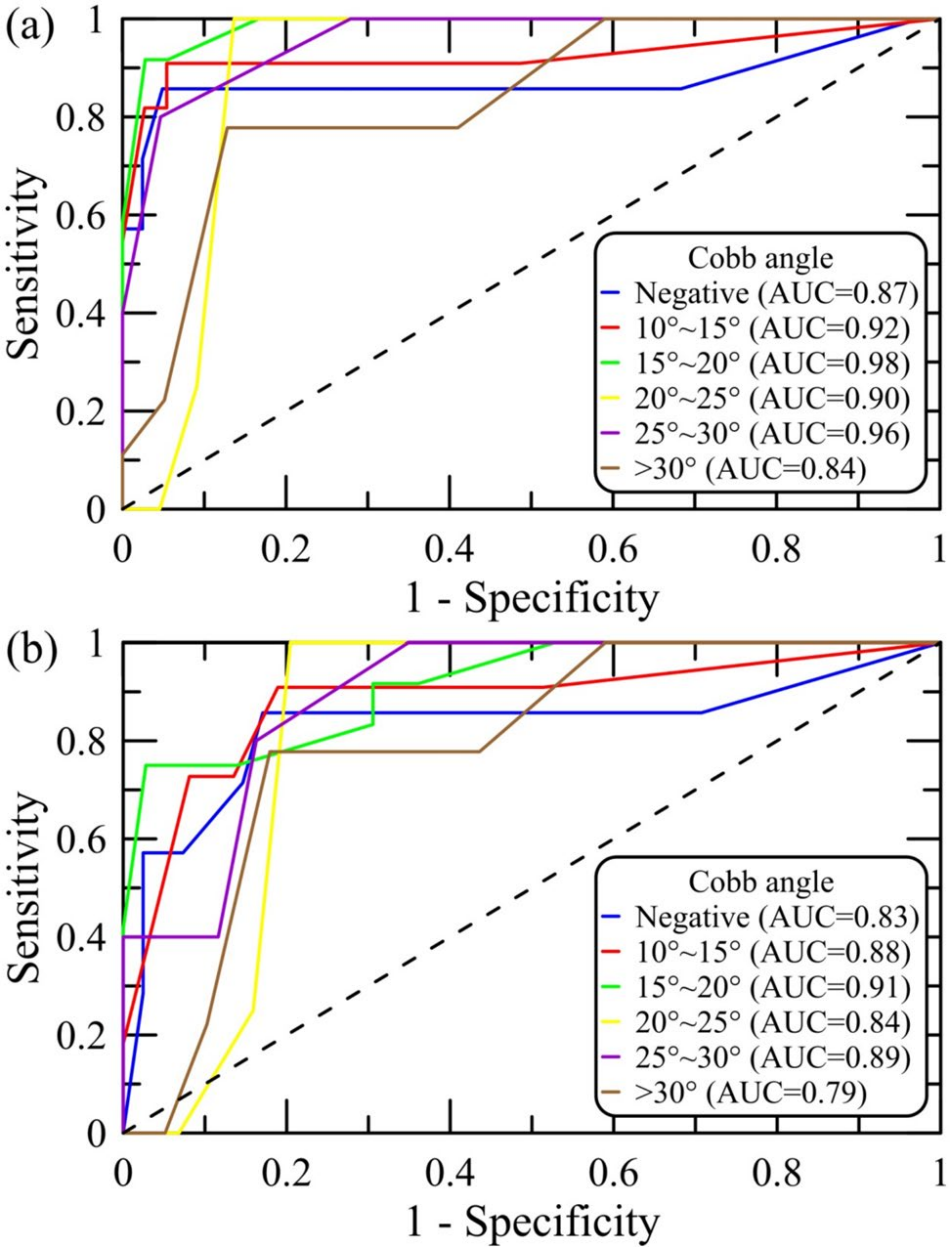
Receiver operating characteristic curves plotted using the convolutional neural networks for **a** Adam’s forward bend test and **b** stand test

Receiver operating characteristic (ROC) analysis was conducted to evaluate asymmetry severity and predict Cobb angle categories: < 10°, 10°–15°, 15°–20°, 20°–25°, 25°–30°, and > 30°. Figure 7 presents ROC curves generated by CNNs for both Adam’s forward bend and standing tests. Diagnostic performance was quantified by the area under the ROC curve (AUC), interpreted as: no discrimination (AUC = 0.5), acceptable (0.7 ≤ AUC < 0.8), excellent (0.8 ≤ AUC < 0.9), and outstanding (AUC ≥ 0.9). Clinically, a Cobb angle > 10° indicates idiopathic scoliosis. For cases exceeding this threshold, the Adam’s forward bend test demonstrated markedly higher discriminative ability, attributable to the enhanced visibility of the spinal hump compared with the upright posture.

Key diagnostic metrics, including positive predictive value (PPV), negative predictive value (NPV), accuracy, positive likelihood ratio (PLR), and negative likelihood ratio (NLR), were computed for both postures (Table 1). The Adam’s forward bend test achieved 89–99% accuracy, outperforming the standing test (83–96%). For the 10°–15° Cobb angle range, it yielded a PPV of 99%, NPV of 95%, accuracy of 96%, PLR of 82%, and NLR of 18%, underscoring its strong diagnostic capability. Overall, these results demonstrate that the Adam’s forward bend test consistently surpasses the standing test in Cobb angle screening, offering superior accuracy and reliability for distinguishing postural asymmetries from true structural scoliosis.

**Table 1:** Experimental indicators for evaluating Cobb angle

Cobb angle	Adam’s forward bend test					Stand test				
	PPV (%)	NPV (%)	Accuracy (%)	PLR (%)	NLR (%)	PPV (%)	NPV (%)	Accuracy (%)	PLR (%)	NLR (%)
Negative	72	98	94	91	14	69	95	88	73	37
10°–15°	99	95	96	82	18	83	97	94	96	10
15°–20°	99	98	99	94	6	92	97	96	94	9
20°–25°	71	98	94	78	26	66	98	92	80	27
25°–30°	83	97	95	73	29	67	98	94	84	21
> 30°	76	93	89	76	30	62	92	83	81	35

*PPV* positive predictive value, *NPV* negative predictive value, *PLR* positive likelihood ratio, *NLR* negative likelihood ratio

**Fig. 8:**
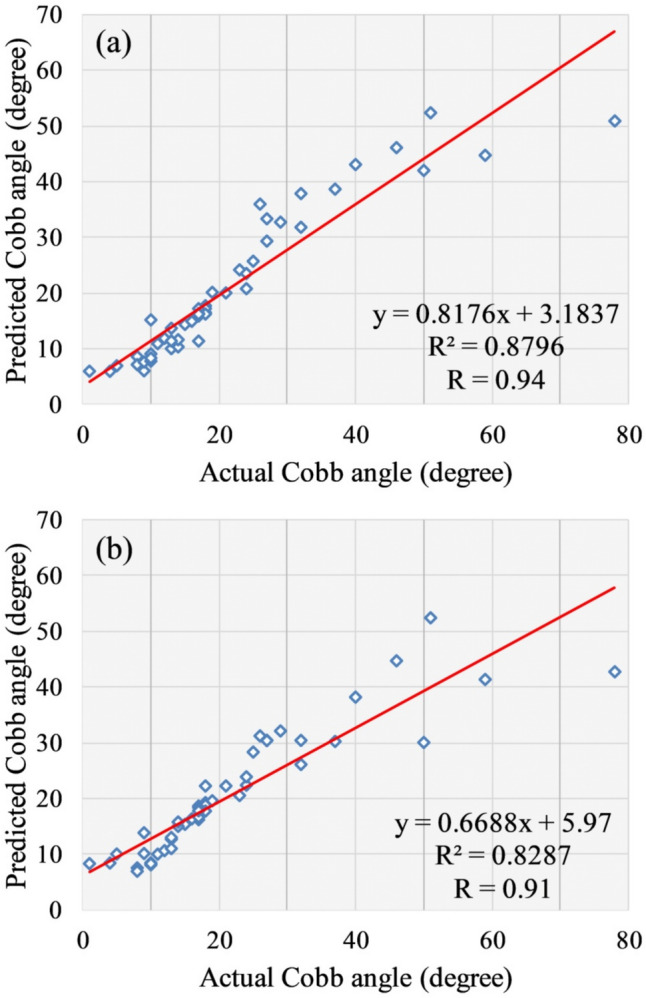
Correlation between the predicted Cobb angle and the actual Cobb angle in total number of subjects for **a** Adam’s forward bend test and **b** stand test

Figure 8 depicts the correlation between predicted and actual Cobb angles for both Adam’s forward bend and standing tests. Pearson’s correlation analysis revealed coefficients of 0.94 and 0.91, with corresponding coefficients of determination (*R*^2^) of 0.8796 and 0.8287, respectively, indicating strong agreement within the clinically relevant 10°–30° range. The Adam’s forward bend test consistently outperformed the standing test in correlation strength. Predictive performance was further assessed using mean absolute error (MAE) and root mean square error (RMSE) across ten repeated validation trials (Table 2). For the Adam’s forward bend test, the MAE and RMSE were 3.9° and 7.3°, respectively, while the standing test yielded 4.5° and 8.9°. The minimal variation across repeats confirmed the system’s stability.

**Table 2:** Mean absolute error (MAE) and root mean square error (RMSE) in the validation with 10 repeats

	Adam’s forward bend test		Stand test	
	MAE (degree)	RMSE (degree)	MAE (degree)	RMSE (degree)
1	3.0	5.4	4.7	9.0
2	3.3	8.1	4.6	9.3
3	4.3	7.9	3.0	6.8
4	4.3	7.7	3.4	7.3
5	4.2	7.6	4.5	8.1
6	4.5	8.1	4.9	9.3
7	3.3	5.9	4.5	8.7
8	4.3	8.0	4.7	9.9
9	3.6	6.6	5.4	10.0
10	3.8	7.3	5.0	10.0
Mean	3.9	7.3	4.5	8.9

## Discussion

This study presents a radiation-free system for scoliosis assessment that integrates fringe projection profilometry with deep learning-based Cobb angle prediction. The results demonstrate that 3D surface reconstruction using a seven-step phase-shifting algorithm provides high-fidelity representations of spinal asymmetry, allowing accurate Cobb angle estimation with strong correlation to radiographic measurements (*r* = 0.94 for the Adam’s forward bend test). The superior diagnostic performance of the Adam’s test compared with the standing posture highlights the clinical importance of posture selection in maximizing surface-based asymmetry detection. Compared with conventional optical methods, such as back photography, Moiré topography, and point cloud-based surface analysis [15, 19, 28], the proposed fringe projection approach offers distinct advantages in both precision and efficiency. Traditional Moiré topography systems often suffer from limited spatial resolution, sensitivity to lighting conditions, and difficulties in reconstructing fine curvature details, especially in patients with mild deformities [10–12]. In contrast, fringe projection profilometry enables dense 3D reconstruction with sub-millimeter accuracy and rapid acquisition, substantially improving surface feature extraction and curvature quantification. This enhanced resolution directly contributes to the superior predictive precision achieved by the proposed CNN-based model. Clinically, combining this imaging modality with the Adam’s forward bend test allows rapid, noninvasive assessment of spinal asymmetry under conditions that accentuate the rib hump and paraspinal contour, key indicators of structural scoliosis. The strong correlation between predicted and radiographic Cobb angles, particularly within the 10°–30° range, aligns with prior studies demonstrating the feasibility of optical surface topography as a surrogate for radiographic evaluation [16–19]. However, unlike previous systems relying primarily on heuristic feature extraction or handcrafted geometric indices, this study employed a data-driven convolutional neural network trained directly on reconstructed surface maps. This approach allows the model to learn subtle, high-dimensional morphological cues associated with underlying spinal deformities, enhancing its generalizability beyond traditional regression-based methods.

Despite these promising findings, several limitations warrant discussion. First, the sample size remains limited, particularly for patients with severe scoliosis (Cobb angle > 40°), which restricts subgroup statistical power and may affect generalizability. In early scoliosis detection, this imbalance may introduce potential misclassification when extrapolating performance beyond mild to moderate curves. Second, although the system demonstrated strong performance in controlled imaging conditions, clinical deployment may face challenges related to patient motion, clothing artifacts, and variable lighting environments. Third, the deep learning model’s training data were derived from static postures; dynamic assessments during motion could provide additional insights into functional asymmetries and compensation mechanisms. Lastly, while this study validates the feasibility of non-invasive Cobb angle estimation, radiography remains indispensable for comprehensive evaluation, including vertebral rotation and sagittal balance. In future work, we plan to obtain new IRB approval to expand subject recruitment and increase the diversity of the dataset, enabling more rigorous model validation across varying scoliosis severities and body morphologies. Integrating multimodal inputs, such as depth maps, reflectance information, and clinical metadata, may further enhance prediction accuracy. Ultimately, continued refinement of the system could support broader clinical translation for safe, radiation-free scoliosis assessment, particularly for longitudinal monitoring and early-stage detection in pediatric populations.

In this proof-of-concept study, we developed and validated a radiation-free system that integrates fringe projection profilometry with deep learning for automated Cobb angle estimation. The current prototype, assembled for preliminary evaluation, was constructed at an approximate cost of 3,000 USD, including the camera, projector, computer, logic controller, metal frame, and associated machining components. Although this configuration is suitable for controlled testing, it reflects an early developmental stage. Based on the study outcomes, further optimization of hardware configuration and system integration is expected to reduce the total system cost to below 1,500 USD. In parallel, future design efforts will focus on hardware miniaturization and improved integration to enhance portability, which is an important consideration for potential use in school or community-based settings. These refinements are anticipated to improve feasibility while maintaining measurement performance. At the current stage, trained personnel are still required for system operation and data verification, underscoring the preliminary nature of this technology. From a clinical perspective, we agree that an important and appropriate application of the proposed radiation-free system is for follow-up visits, where repeated assessment of spinal alignment is often necessary. In this context, the method may assist in longitudinal monitoring of curve progression and help reduce cumulative radiation exposure associated with repeated radiographic examinations. The system may also serve as a preliminary screening or adjunctive assessment tool when clinically appropriate. Importantly, the proposed technique is not intended to replace radiographic imaging, which remains essential for definitive diagnosis and clinical decision-making. Rather, its role is to complement standard X-ray examinations by reducing imaging frequency when possible, while preserving patient safety and diagnostic reliability. Compared with conventional surface topography techniques, such as Moiré imaging, back photography, and point cloud reconstruction, fringe projection profilometry provides higher spatial resolution and faster data acquisition, enabling more detailed characterization of spinal surface morphology.

## Conclusion

In conclusion, this study presents a rapid, radiation-free approach for evaluating spinal asymmetry and estimating the Cobb angle from three-dimensional back surface imaging. By combining a fully automated, high-speed digital fringe projection profilometry system with a convolutional neural network regression model, spinal curvature could be assessed without manual landmark identification or specialist-dependent measurement. When applied in conjunction with the Adam’s forward bend test, the proposed method demonstrated strong agreement with radiographic measurements, achieving a Pearson correlation coefficient of 0.94. These findings support the feasibility of using surface-based optical imaging as a complementary tool for scoliosis assessment, with the potential to reduce reliance on repeated radiographic examinations and associated radiation exposure in adolescents. Importantly, this technique is not intended to replace radiographic imaging, which remains essential for definitive diagnosis and clinical decision-making, but rather to assist in noninvasive monitoring and follow-up evaluations when appropriate. While the results are encouraging, the current study represents a proof-of-concept with a limited cohort. Future work will focus on expanding patient populations, improving model generalization, refining posture alignment automation, and further validating clinical robustness before broader clinical adoption is considered.

## Data Availability

The data that support the findings of this study are available from the corresponding author upon reasonable request.
